# The distribution and the relationship of HPV subtypes infection with pregnancy outcomes

**DOI:** 10.1038/s41598-025-94563-4

**Published:** 2025-03-24

**Authors:** Yingying Lin, Zhiwei Chen, Xinye Zheng, Yuanjie Qi, Li Xie, Yulong Zhang, Hua Li

**Affiliations:** 1 Department of Healthcare, Fujian Maternity and Child Health Hospital, College of Clinical Medicine for Obstetrics & Gynecology and Pediatrics, Fujian Medical University, Fuzhou, China; 2 Department of Obstetrics and Gynecology, Fujian Maternity and Child Health Hospital, College of Clinical Medicine for Obstetrics & Gynecology and Pediatrics, Fujian Medical University, Fuzhou, China

**Keywords:** Human papillomavirus (HPV), Pregnancy, Maternal outcomes, Neonatal outcomes, HPV vaccine, Infectious diseases, Outcomes research, Public health

## Abstract

Human Papillomavirus (HPV) infection is a significant public health concern, particularly during pregnancy. The impact of HPV infection during pregnancy on maternal and child health is a growing concern. However, research on infection rate and pregnancy outcomes of nine-valent vaccine-covered and non-covered high-risk HPV subtypes during pregnancy remains limited. A retrospective cohort study involved 7110 pregnant women who underwent HPV testing in Fujian Provincial Maternity and Child Health Hospital, Affiliated Hospital of Fujian Medical University (FMCH) from January 2021 to December 2022. Multiple logistic regression analyses investigated the association between HPV infections and maternal and neonatal outcomes. The study identified that the overall HPV infection rate in pregnant women was 19.9%. The HPV types 52, 16, 58, 42, and 51 were the most common among the participants. Infections with nine-valent HPV vaccine-covered high-risk subtypes significantly increased the risk of preterm premature rupture of membranes (PPROM) (OR = 1.29; 95% CI: 1.08–1.55) and cesarean section (OR = 1.25; 95% CI: 1.06–1.49). Co-infections with both nine-valent vaccine-covered and non-covered high-risk HPV subtypes elevated the risks of cesarean section (OR = 1.59; 95% CI: 1.11–2.27) and hypertensive disorders of pregnancy (OR = 2.73; 95% CI: 1.44–5.15). Additionally, these infections significantly increased the risk of small for gestational age (SGA) infants, with the group IV (OR = 2.07; 95% CI: 1.21–3.54) having a higher risk than the group III (OR = 1.33; 95% CI: 0.17–1.65). Moreover, those neonates who were born to mothers with HPV co-infections had a significantly higher risk of ICU admission (OR = 1.58; 95% CI: 1.01–2.49). This study revealed the significant association between specific HPV infections during pregnancy and adverse maternal and neonatal outcomes. These findings underscore the importance of monitoring HPV infections during pregnancy and suggest that an enhanced rate of the nine-valent HPV vaccine and strengthen the development and application of the non-nine-valent HPV vaccine could improve maternal–fetal health outcomes.

## Introduction

Human papillomavirus (HPV) is one of the most common sexually transmitted pathogens. Continuous HPV infection can lead to premalignant lesions in the uterus, vagina, anus, penis, and throat and malignant lesions^1^. About 70% of cervical cancer and HPV-related genital and anal cancers were caused by HPV-16/18, while low-risk HPV-6/11 can cause 90% of condyloma^2^. More than half of sexually active adults were infected with at least one HPV subtype, but most HPV infections were transient and do not cause related symptoms or health-related problems. If HPV infection continues, it might lead to premalignant and malignant lesions and was not easily recognized due to the absence of typical clinical symptoms^3^. Pregnant women represent a unique population in the study of HPV infection due to physiological changes during pregnancy that may influence the natural history of HPV^4^. Studies have presented that pregnancy can alter the immune response, potentially affecting the persistence and clearance of HPV infections^5^. The phenotypic characteristics of HPV infection, such as viral load, genotype distribution, and persistence, have been well studied in non-pregnant populations^6^. However, there is limited data on the phenotypic characteristics of HPV infection in pregnant women, a gap that this study aims to address. Thus, it is essential to underscore the importance of understanding the distribution and impact of HPV infection in pregnant women to inform better clinical management and preventive measures.

In recent years, with the promotion and application of HPV vaccines, certain progress has been made in the prevention and control of HPV infections. However, a recent survey published by the Weekly report of the China Center for Disease Control and Prevention shows that the coverage rate of HPV vaccine in China remain considerably below global averages and WHO targets^7^. By 2022, first-dose coverage stood at only 10.15%, with full-series coverage at 6.21%^7^. As a result, HPV vaccination rates are lower among women of childbearing age. Due to the immunosuppressive effects of pregnancy hormones, the risk of HPV infection in pregnant women is increased. Therefore, understanding the impact of the HPV infection on the outcome of maternal pregnancy is a critical issue. Unfortunately, there are limited studies about different types of HPV infection on maternal pregnancy outcomes.

Several studies have been conducted to evaluate the effect of HPV infection during pregnancy on pregnancy outcomes. However, the results of these studies have been inconsistent. Some studies have found that HPV infection during pregnancy may be associated with various adverse pregnancy outcomes, including preterm birth^8^, PPROM^9^, fetal growth restriction^10^, and SGA^11^, or even fetal death^12^. However, the study by YaJun Liu et al.^13^did not find a significant correlation. Similarly, some research revealed that HPV infection increased the risk of preterm birth and spontaneous abortion^14,15^. While other studies suggested that this association was not significant^16,17^. Similarly, findings regarding the relationship between HPV infection and PPROM, cesarean delivery, and SGA remain controversial.

Given these inconsistent research findings, further exploration of the impact of HPV infection during pregnancy on maternal and fetal outcomes holds significant clinical importance. There are three human HPV vaccines marketed in China. The nine-valent HPV vaccine covers multiple HPV types commonly found in China, and it is the most significant demand for the HPV vaccine. Before 2023, the age of vaccination for the nine-valent vaccine in China is from 16 to 26 years old. Coupled with tight supply, the vast majority of women of childbearing age did not receive the nine-valent HPV vaccine before becoming pregnant.Therefore, this study aims to analyze the situation and clinical characteristics of HPV infection in domestic pregnant women and investigate the effects of covered and non-covered high-risk HPV subtypes of 9-valent HPV vaccine on pregnant women and newborns, providing valuable references for further research and clinical practice, thereby improving maternal and neonatal outcomes, and offering a scientific basis for future vaccine development and public health policies.

## Material and methods

### The participant’s samples

This study was a retrospective cohort study. We use electronic medical records to collect and analyze the clinical data of 8054 pregnant women who underwent HPV examination in Fujian Provincial Maternity and Child Health Hospital, Affiliated Hospital of Fujian Medical University (FMCH) and gave birth in the hospital from January 2021 to December 2022. Based on the inclusion and exclusion criteria, we finally included 7110 pregnant women for the analysis. HPV typing data, essential clinical information and pregnancy outcomes of participants were collected. The Hospital Ethics Committee of Fujian Provincial Maternity and Children’s Hospital, an affiliated hospital of Fujian Medical University, approved the study (2024KY161). All research was performed in accordance with relevant guidelines/regulations. All Research have been performed in accordance with the Declaration of Helsinki.

All the study participants must meet the following inclusion criteria: (1) the gestational week ≥ 20 weeks of delivery; (2) singleton pregnancy; (3) no previous history of cervical surgery; (4) regular obstetric examination and delivery in Fujian Provincial Maternity and Child Health Hospital, Affiliated Hospital of Fujian Medical University; (5) no sexual life or history of vaginal suppositories within three days of examination. Exclusion criteria were as follows: (1) pregnancy with severe internal and surgical diseases, including pregnancy with serious heart disease, kidney disease, liver disease, encephalopathy and so on; (2) patients with malignant tumours; (3) psychiatric history; (4) incomplete critical data, such as missing HPV test results, records of major pregnancy outcomes, or important demographic information. This study used the International Classification of Diseases (ICD-10) for disease definition.

### The Acquisition and management of specimens for HPV testing

All pregnant women underwent HPV testing in the second trimester, and Exfoliated cervical cells from each pregnant woman were collected by cell brush and persevered in 2 ml vials containing preservation solution for HPV testing. All samples were stored at − 20 °C before DNA extraction.

### HPV genotype testing

The HPV detection is based on the PCR-reverse point hybridization technique. The PCR-RDB HPV genotyping kit for 23 types (Yaneng Bioscience Co., Ltd., China)^18^was used for this study. It detects 18 HR-HPV types, including HPV 16, 18, 31, 33, 35, 35, 39, 49, 55, 51, 52, 53, 56, 58, 59, 66, 68, 73, 82 and 83 and 5 LR-HPV types including HPV 6, 11, 42, 43 and81. All testing procedures followed the manufacturer’s instructions provided with the kit^19^.

### Study groups

This study was grouped according to different subtypes of HPV infection, focusing on the impact of high-risk HPV subtypes covered by the nine-valent HPV vaccine on pregnancy outcomes. The control group are pregnant women without HPV infection; group I means low risk HPV infection (HPV6, 11, 42, 43, 44, 81, 83); group II means infection only with nine-valent HPV vaccine covered high-risk HPV subtypes (HPV16, 18, 31, 33, 45, 52, and 58); group III means infection with non-nine-valent HPV vaccine covered high-risk HPV subtypes (HPV 35, 39, 51, 53, 56, 59, 66, 68, 73, and 82) ; group IV means co-infection with nine-valent HPV vaccine covered high-risk HPV subtypes and non-nine-valent HPV vaccine covered high-risk HPV subtypes.

### Statistics

The R software 4.2.2 was used for the statistical analysis. Measurement data are shown as means ± standard deviation, and group analysis was compared using ANOVA. Count data is expressed as a rate; χ^2^ test was used in group analysis. Multiple logistic regression analysis was used to evaluate the effect of nine-valent HPV vaccine-covered HPV subtype infection and non-nine-valent HPV vaccine infection on pregnancy outcomes. A *P* < 0.05 indicated that the differences were statistically significant.

## Result

### Essential characteristic of the study cohort

Finally, 7110 pregnant women were included in this study. All patients were divided into five groups according to their HPV infection status, Group I (n = 216), Group II (n = 655), Group III (n = 414), Group IV (n = 135), and control Group (n = 5690).

The primary clinical information for all patients is shown in Table 1. There was no statistical difference in the clinical basis information of the five groups in this study. The mean age of the study cohort was 31.1 ± 4.3 years. Of all pregnant women, 2753 (38.7%) had once gravity, 2160 (30.4%) twice gravity, and 2195 (30.9%) three times gravity. Also, 4271 (60.1%) 0–1 parity, and 2839 (39.9%) twice parity. Of the 7110 newborns, 3716 (52.3%) were males and 3394 (47.7%) were females. And then, the mean gestational age at delivery was 38.6 ± 2.1 weeks. The mean birth weight was 3237.9 ± 516.5 g, and the mean baby height was 49.3 ± 2.6 cm.

**Table 1:** Clinical characteristics of the study cohort.

**Variables**	Total (n = 7110)	**HPV infection**					***p***
		Control (n = 5690)	I (n = 216)	II (n = 655)	III (n = 414)	IV (n = 135)	
Maternal age (years)	31.1 ± 4.3	31.2 ± 4.2	30.7 ± 4.6	30.7 ± 4.2	31.0 ± 4.7	30.3 ± 4.6	0.005
Gravidity n (%)							
1	2753 (38.7)	2183 (38.4)	96 (44.7)	258 (39.4)	157 (37.9)	59 (43.7)	0.148
2	2160 (30.4)	1771 (31.1)	54 (25.1)	179 (27.3)	117 (28.3)	39 (28.9)	
3	2195 (30.9)	1735 (30.5)	65 (30.2)	218 (33.3)	140 (33.8)	37 (27.4)	
Parity n (%)							
0–1	4271 (60.1)	3396 (59.7)	140 (64.8)	402 (61.4)	250 (60.4)	83 (61.5)	0.562
2	2839 (39.9)	2294 (40.3)	76 (35.2)	253 (38.6)	164 (39.6)	52 (38.5)	
Baby sex n (%)							
Male	3716 (52.3)	3020 (53.1)	103 (47.7)	314 (47.9)	205 (49.5)	74 (54.8)	0.043
Female	3394 (47.7)	2670 (46.9)	113 (52.3)	341 (52.1)	209 (50.5)	61 (45.2)	
GA at delivery (weeks)	38.6 ± 2.1	38.6 ± 2.1	38.7 ± 2.0	38.5 ± 2.3	38.6 ± 2.4	38.8 ± 1.9	0.313
Birth weight (g)	3237.9 ± 516.5	3239.1 ± 513.8	3195.9 ± 488.8	3215.3 ± 540.1	3267.6 ± 527.0	3269.9 ± 523.0	0.330
Baby height (cm)	49.3 ± 2.6	49.3 ± 2.6	49.1 ± 2.8	49.1 ± 2.7	49.3 ± 3.0	49.4 ± 2.2	0.514

Continuous variables are presented as mean ± SD (range), ANOVA was used for comparison, and categorical variables as n (%), Chi-square test was used for comparison.

GA, gestational age; HPV, Human papillomavirus; Control group means without HPV infection; Group I means low risk HPV infection; Group II means infection with nine-valent HPV vaccine covered high-risk HPV subtypes; Group III means infection with non-nine-valent HPV vaccine covered high-risk HPV subtypes; Group IV means co-infection with nine-valent HPV vaccine covered high-risk HPV subtypes and non-nine-valent HPV vaccine covered high-risk HPV subtypes.

### Distribution of infection rates of HPV subtypes

**Fig. 1:**
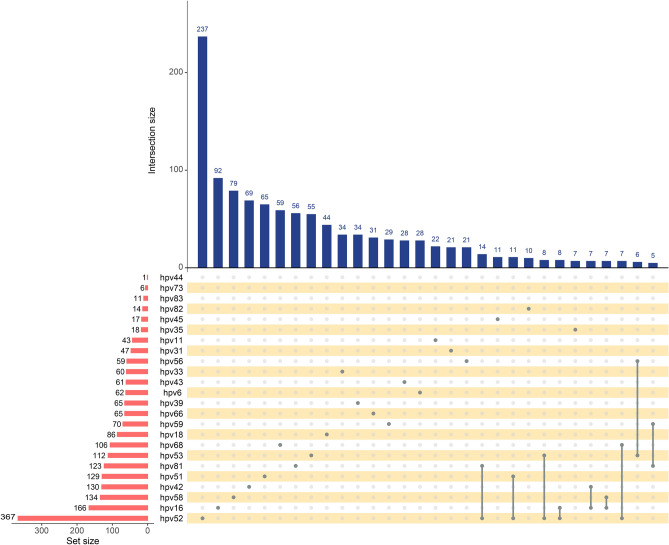
HPV infection in the population HPV single infection and co-infection in the total population.

The infection of different HPV subtypes in all participants is shown in Fig. 1. Our results suggested that most pregnant women are HPV negative, followed by 9.2% of pregnant women with low-risk HPV infection (group I ), group II (5.8%), group III (3.0%), and group IV (1.9%). We then analyzed the specific HPV subtypes and found that HPV 52, 16, 58, 42 and 51 were the five most common infection types. The above HPV subtypes were high-risk HPV infections except HPV 42. Also, most of these HPV infections are isolated infections. Co-infection with HPV 52 and HPV 81, HPV 52 and HPV 51 is the most common type of multiple infection.

Distribution of all HPV subtypes infection in the population.

**Fig. 2:**
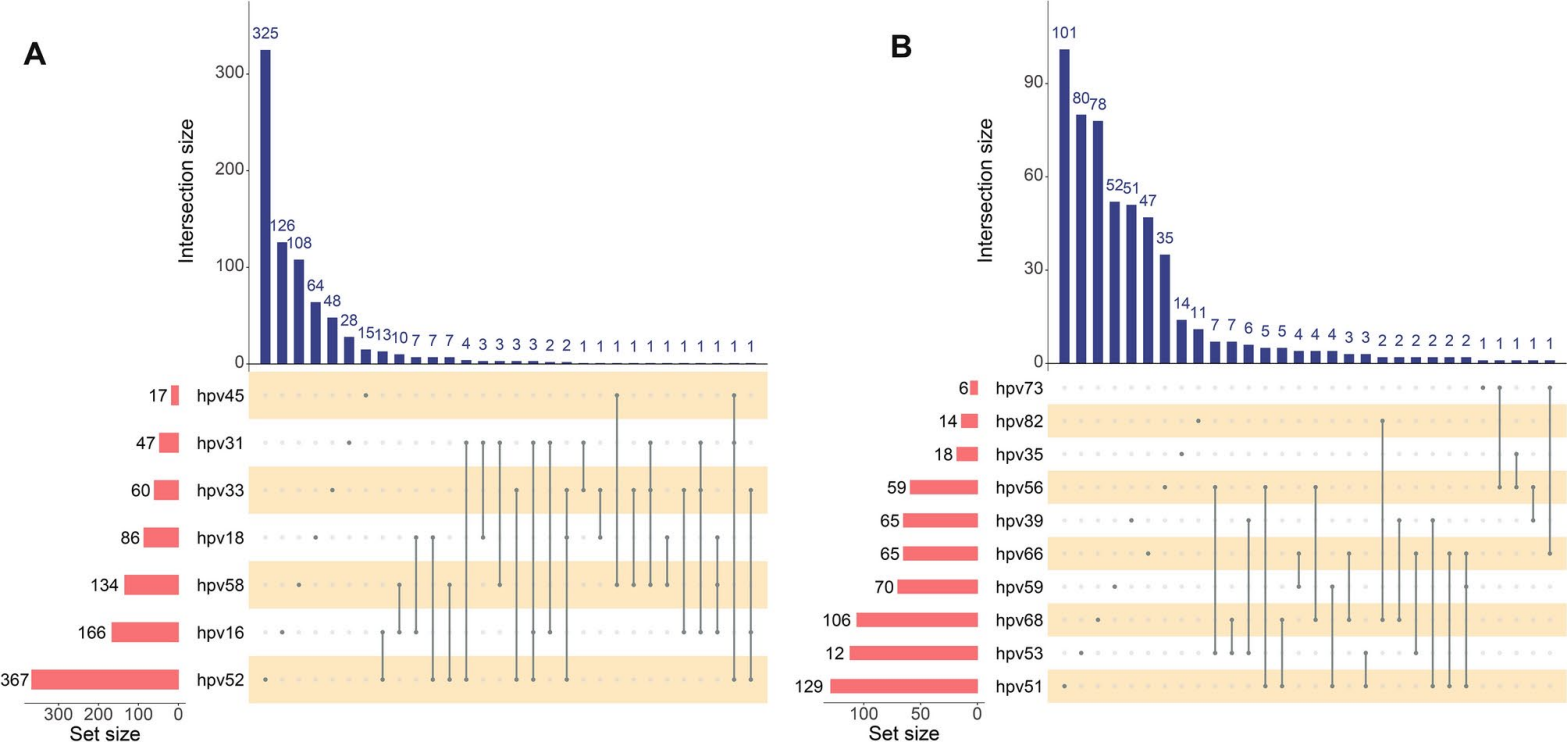
Distribution of nine-valent vaccine-covered and non-covered high-risk HPV subtypes **A**: Distribution of nine-valent vaccine-covered high-risk HPV subtypes infection; **B**: Distribution of non- nine-valent vaccine-covered high-risk HPV subtypes infection.

Furthermore, the distribution of nine-valent HPV vaccine-covered high-risk HPV subtypes and non-nine-valent HPV vaccine-covered high-risk HPV subtypes is shown in Fig. 2. Among them, the most common in nine-valent HPV vaccine-covered high-risk HPV subtypes were HPV 52 and 16, followed by 58, 18 and 33. Co-infection with HPV 16 and HPV 52, HPV 16 and HPV 58 were the most common type of multiple infection. As for non-nine-valent HPV vaccine-covered high-risk HPV subtypes, HPV51, 53, 68, 59 and 66 were the most common infections. Co-infection with HPV 53 and 56, HPV 53 and 68 were the most common multiple infection.

### Correlation between HPV subtype infection and maternal outcomes

Next, we continued to analyze the relationship between HPV subtype infection and maternal pregnancy outcomes. Our findings indicated that HPV infection increases the risk of HDCP (OR = 1.40, (95%CI) (1.04–1.87)) and cesarean section (OR = 1.19, (95%CI) (1.05–1.35)). Further subgroup analysis showed that the incidence of both PPROM and cesarean section is significantly higher in the group II , suggesting that nine-valent HPV vaccine-covered HPV subtypes infection increases the risk of PPROM (OR = 1.29, (95%CI) (1.08–1.55)) and cesarean section (OR = 1.25, (95%CI) (1.06 to 1.49)). Besides, The results further indicated that pregnant women with co-infection with nine-valent HPV vaccine-covered HPV subtypes and non-nine-valent HPV vaccine-covered HPV subtypes had a cesarean section (OR = 1.59, (95%CI) (1.11–2.27)). Hypertensive disorders of pregnancy (HDCP) (OR = 2.73, (95%CI) (1.44 to 5.15)) had the highest risk. However, there was no statistical difference in the incidence of HPV infection and these complications, such as gestational diabetes (GDM), placental abruption and postpartum haemorrhage during pregnancy (Table 2).

**Table 2:** Effect of infection with HPV subtypes on maternal outcomes.

Variables	HPV group	N	Event, n(%)	Crude		Adjusted	
				**OR (95%CI)**	***P* value**	**OR (95%CI)**	***P* value**
GDM	Control	5690	1294 (22.7)	1(Ref)		1(Ref)	
	I	216	49 (22.7)	1.00 (0.72 ~ 1.38)	0.984	1.03 (0.74 ~ 1.44)	0.849
	II	655	123 (18.8)	0.79 (0.64 ~ 0.96)	0.021	0.82 (0.66 ~ 1.01)	0.060
	III	414	87 (21.0)	0.90(0.71 ~ 1.15)	0.418	0.89 (0.70 ~ 1.15)	0.384
	IV	135	27 (20.0)	0.85 (0.55 ~ 1.30)	0.453	0.92 (0.59 ~ 1.42)	0.704
	HPV infection	1420	286 (20.1)	0.86 (0.74 ~ 0.99)	0.035	0.88 (0.76 ~ 1.02)	0.092
HDCP	Control	5690	187 (3.3)	1(Ref)		1(Ref)	
	I	216	11 (5.1)	1.58 (0.85 ~ 2.95)	0.151	1.61 (0.86 ~ 3.01)	0.135
	II	655	28 (4.3)	1.31 (0.88 ~ 1.97)	0.187	1.36 (0.91 ~ 2.05)	0.138
	III	414	13 (3.1)	0.95 (0.54 ~ 1.69)	0.872	0.95 (0.54 ~ 1.68)	0.860
	IV	135	11 (8.1)	2.61 (1.39 ~ 4.92)	0.003*	2.73 (1.44 ~ 5.15)	0.002*
	HPV infection	1420	63 (4.4)	1.37 (1.02 ~ 1.83)	0.036	1.40 (1.04 ~ 1.87)	0.025*
PPROM	Control	5690	1448 (25.4)	1(Ref)		1(Ref)	
	I	216	58 (26.9)	1.08 (0.79 ~ 1.46)	0.642	1.06 (0.78 ~ 1.45)	0.706
	II	655	200 (30.5)	1.29 (1.08 ~ 1.54)	0.005*	1.29 (1.08 ~ 1.55)	0.005*
	III	414	94 (22.7)	0.86 (0.68 ~ 1.09)	0.215	0.86 (0.67 ~ 1.09)	0.203
	IV	135	35 (25.9)	1.03 (0.69 ~ 1.51)	0.900	1.01 (0.68 ~ 1.49)	0.977
	HPV infection	1420	387 (27.3)	1.10 (0.96 ~ 1.25)	0.164	1.09 (0.96 ~ 1.25)	0.186
Placental abruption	Control	5690	145 (2.5)	1(Ref)		1(Ref)	
	I	216	3 (1.4)	0.54 (0.17 ~ 1.70)	0.292	0.53 (0.17 ~ 1.70)	0.289
	II	655	11 (1.7)	0.65 (0.35 ~ 1.21)	0.177	0.64 (0.34 ~ 1.20)	0.162
	III	414	12 (2.9)	1.14 (0.63 ~ 2.07)	0.664	1.22 (0.67 ~ 2.22)	0.521
	IV	135	3 (2.2)	0.87 (0.27 ~ 2.76)	0.812	0.96 (0.30 ~ 3.07)	0.951
	HPV infection	1420	29 (2)	0.80 (0.53 ~ 1.19)	0.270	0.81 (0.54 ~ 1.22)	0.321
Postpartum hemorrhage	Control	5690	471 (8.3)	1(Ref)		1(Ref)	
	I	216	18 (8.3)	1.01 (0.62 ~ 1.65)	0.977	1.04 (0.63 ~ 1.70)	0.887
	II	655	50 (7.6)	0.92 (0.68 ~ 1.24)	0.570	0.95 (0.70 ~ 1.29)	0.760
	III	414	38 (9.2)	1.12 (0.79 ~ 1.58)	0.522	1.11 (0.78 ~ 1.57)	0.567
	IV	135	9 (6.7)	0.79 (0.40 ~ 1.57)	0.502	0.84 (0.42 ~ 1.67)	0.618
	HPV infection	1420	115 (8.1)	0.98 (0.79 ~ 1.21)	0.826	1.00(0.81 ~ 1.24)	0.991
Cesarean delivery	Control	5690	2029 (35.7)	1(Ref)		1(Ref)	
	I	216	77 (35.6)	1.00 (0.75 ~ 1.33)	0.997	1.02 (0.76 ~ 1.38)	0.875
	II	655	257 (39.2)	1.17 (0.99 ~ 1.38)	0.071	1.25 (1.06 ~ 1.49)	0.010*
	III	414	154 (37.2)	1.07 (0.87 ~ 1.31)	0.528	1.07 (0.86 ~ 1.33)	0.526
	IV	135	59 (43.7)	1.40 (0.99 ~ 1.98)	0.055	1.59 (1.11 ~ 2.27)	0.011*
	HPV infection	1420	547 (38.5)	1.13 (1.00 ~ 1.27)	0.045	1.19 (1.05 ~ 1.35)	0.006*

Adjusted OR means adjusted for maternal age, Gravidity, Parity and Baby sex. *P < 0.05.

GDM, gestational diabetes; HDCP, hypertensive disorder complicating pregnancy; PPROM, preterm premature rupture of membranes; Control group means without HPV infection; group I means low risk HPV infection; group II means infection with nine-valent HPV vaccine covered high-risk HPV subtypes; group III means infection with non-nine-valent HPV vaccine covered high-risk HPV subtypes; group IV means co-infection with nine-valent HPV vaccine covered high-risk HPV subtypes and non-nine-valent HPV vaccine covered high-risk HPV subtypes.

### Correlation between HPV subtype infection and neonatal outcomes

To further investigate whether HPV subtype infection affected pregnancy outcomes in newborns. The results of the multiple logistic regression analysis demonstrated that nine-valent HPV vaccine-covered high-risk HPV subtypes infection and co-infection HPV subtypes can significantly increase the risk of small for gestational age (SGA). Group IV(OR = 2.07, (95%CI) (1.21–3.54)) had a higher risk than the group III(OR = 1.33, (95%CI) (0.17–1.65)). In addition, we found that the risk of ICU admission in maternal with co-infection HPV subtypes significantly increased (OR = 1.58, (95%CI) (1.01–2.49) (Table 3).

**Table 3:** Effect of infection with HPV subtypes on neonatal outcomes.

	**HPV group**	**N**	**Event, n(%)**	**Crude**		**Adjusted**	
				**OR (95%CI)**	***P* value**	**OR (95%CI)**	***P* value**
Premature birth	Control	5690	423 (7.4)	1(Ref)		1(Ref)	
	I	216	15 (6.9)	0.93 (0.54 ~ 1.58)	0.788	0.88 (0.50 ~ 1.55)	0.654
	II	655	64 (9.8)	1.35 (1.02 ~ 1.78)	0.034*	1.32 (0.99 ~ 1.77)	0.058
	III	414	27 (6.5)	0.87 (0.58 ~ 1.30)	0.493	0.92 (0.62 ~ 1.38)	0.696
	IV	135	12 (8.9)	1.21 (0.67 ~ 2.22)	0.526	1.35 (0.74 ~ 2.47)	0.328
	HPV infection	1420	118 (8.3)	1.13 (0.91 ~ 1.4)	0.266	1.14 (0.91 ~ 1.42)	0.256
SGA	Control	5690	348 (6.1)	1(Ref)		1(Ref)	
	I	216	20 (9.3)	1.56 (0.97 ~ 2.51)	0.064	1.47 (0.91 ~ 2.37)	0.113
	II	655	44 (6.7)	1.10 (0.80 ~ 1.53)	0.554	1.05 (0.75 ~ 1.45)	0.787
	III	414	9 (2.2)	1.34 (0.18 ~ 1.67)	0.002*	1.33 (0.17 ~ 1.65)	0.001*
	IV	135	16 (11.9)	2.06 (1.21 ~ 3.51)	0.008*	2.07 (1.21 ~ 3.54)	0.008*
	HPV infection	1420	89 (6.3)	1.03 (0.81 ~ 1.30)	0.836	0.98 (0.77 ~ 1.25)	0.891
LGA	Control	5690	377 (6.6)	1(Ref)		1(Ref)	
	I	216	6 (2.8)	0.40(0.18 ~ 0.91)	0.029*	0.43 (0.19 ~ 0.97)	0.043*
	II	655	51 (7.8)	1.19 (0.88 ~ 1.61)	0.269	1.25 (0.92 ~ 1.70)	0.155
	III	414	26 (6.3)	0.95 (0.63 ~ 1.43)	0.795	0.97 (0.64 ~ 1.47)	0.893
	IV	135	11 (8.1)	1.25 (0.67 ~ 2.33)	0.489	1.28 (0.68 ~ 2.39)	0.446
	HPV infection	1420	94 (6.6)	1.00 (0.79 ~ 1.26)	0.989	1.04 (0.82 ~ 1.32)	0.732
LBW	Control	5690	325 (5.7)	1(Ref)		1(Ref)	
	I	216	12 (5.6)	0.97 (0.54 ~ 1.76)	0.923	0.86 (0.45 ~ 1.65)	0.656
	II	655	52 (7.9)	1.42 (1.05 ~ 1.93)	0.023	1.35 (0.98 ~ 1.86)	0.068
	III	414	21 (5.1)	0.88 (0.56 ~ 1.39)	0.587	0.94 (0.60 ~ 1.49)	0.802
	IV	135	11 (8.1)	1.46 (0.78 ~ 2.74)	0.233	1.63 (0.87 ~ 3.05)	0.130
	HPV infection	1420	96 (6.8)	1.2 0(0.95 ~ 1.51)	0.135	1.18 (0.92 ~ 1.51)	0.190
Macrosomia	Control	5690	250 (4.4)	1(Ref)		1(Ref)	
	I	216	5 (2.3)	0.52 (0.21 ~ 1.26)	0.147	0.54 (0.22 ~ 1.32)	0.178
	II	655	21 (3.2)	0.72 (0.46 ~ 1.13)	0.156	0.74 (0.47 ~ 1.17)	0.202
	III	414	13 (3.1)	0.71 (0.40 ~ 1.24)	0.227	0.72 (0.41 ~ 1.26)	0.247
	IV	135	7 (5.2)	1.19 (0.55 ~ 2.57)	0.658	1.20 (0.56 ~ 2.61)	0.639
	HPV infection	1420	46 (3.2)	0.73 (0.53 ~ 1.00)	0.052	0.75 (0.54 ~ 1.03)	0.076
NICU admission	Control	5690	706 (12.4)	1(Ref)		1(Ref)	
	I	216	23 (10.6)	0.84 (0.54 ~ 1.31)	0.441	0.83 (0.54 ~ 1.30)	0.423
	II	655	76 (11.6)	0.93 (0.72 ~ 1.19)	0.553	0.94 (0.73 ~ 1.21)	0.615
	III	414	51 (12.3)	0.99 (0.73 ~ 1.34)	0.958	1.00 (0.74 ~ 1.36)	0.991
	IV	135	24 (17.8)	1.53 (0.98 ~ 2.39)	0.064	1.58 (1.01 ~ 2.49)	0.046*
	HPV infection	1420	174 (12.3)	0.99 (0.83 ~ 1.18)	0.875	1.00 (0.83 ~ 1.19)	0.968

Adjusted OR means adjusted for maternal age, Gravidity, Parity and Baby sex. *P < 0.05.

SGA,small for gestational age; LGA, large for gestational age; LBW, low birth weight infant; NICU, neonatal intensive care unit; Control group means without HPV infection; group I means low risk HPV infection; group II means infection with nine-valent HPV vaccine covered high-risk HPV subtypes; group III means infection with non-nine-valent HPV vaccine covered high-risk HPV subtypes; group IV means co-infection with nine-valent HPV vaccine covered high-risk HPV subtypes and non-nine-valent HPV vaccine covered high-risk HPV subtypes.

## Discussion

HPV infection is a significant public health concern, particularly among women of reproductive age^8^. Given the potential for severe health outcomes, understanding the distribution and impact of HPV infection during pregnancy is crucial. Pregnant women represent a unique population where HPV infection could have implications not only for their maternal health but also for neonatal outcomes. However, there is limited data on the phenotypic characteristics of HPV infection in pregnant women. This study focuses on the distribution of HPV infection among pregnant women and its potential impact on pregnancy outcomes.

In this study, we analyzed data from 7110 pregnant women who underwent HPV testing. The results identified that the overall HPV infection rate in pregnant women was 19.9%. The HPV types 52, 16, 58, 42, and 51 were the most common among the participants. Infections with nine-valent HPV vaccine-covered high-risk subtypes significantly increased the risk of preterm premature rupture of membranes (PPROM) (OR = 1.29; 95% CI: 1.08–1.55) and cesarean section (OR = 1.25; 95% CI: 1.06–1.49). Co-infections with both nine-valent vaccine-covered and non-covered high-risk HPV subtypes elevated the risks of cesarean section (OR = 1.59; 95% CI: 1.11–2.27) and hypertensive disorders of pregnancy (OR = 2.73; 95% CI: 1.44–5.15). The findings of this study are significant as they provide a comprehensive overview of HPV prevalence in this specific population and highlight the potential risks associated with different HPV types during pregnancy.

In our study, HPV 52 is one of the high-risk HPV subtypes frequently detected. It suggested its significant role in HPV-related pathologies during pregnancy. The high prevalence of HPV 52 among pregnant women underscores the necessity for targeted screening and vaccination strategies. Previous studies have also highlighted the association of HPV 52 with adverse pregnancy outcomes, including PPROM and increased cesarean delivery rates^20^. The data from our study corroborate these findings. Moreover, our study results align with existing literature that links HPV 16 to various obstetric complications^21^. These findings are similar to previous research that has identified HPV 58 as a contributor to obstetric complications^22^. The high prevalence of HPV 52, 16, 58 and 42 in our cohort highlights the critical need for effective vaccination programs, such as the nine-valent HPV vaccine, which includes the above HPV subtypes. Our results emphasize the importance of monitoring nine-valent HPV vaccine-covered HPV subtypes of infection during pregnancy to mitigate potential risks to both the mother and the fetus.

Importantly, co-infection with both nine-valent HPV vaccine-covered types and non-nine-valent types was found to significantly increase the risk of cesarean delivery and hypertensive disorders of pregnancy (HDP) in our study. This finding is critical as it highlights the compounded risk posed by multiple HPV infections. Previous studies have shown that co-infections can exacerbate the severity of HPV-related diseases and complicate pregnancy outcomes^23^. These findings highlight the complex interplay between multiple HPV infections and the heightened risk of pregnancy complications, necessitating comprehensive screening and management strategies^24^^.^

Additionally, the study reveals a significant association between HPV infections and the risk of delivering small for gestational age (SGA) infants. Specifically, infections with nine-valent vaccine-covered subtypes and co-infections significantly increase the risk of SGA, with group IV (OR = 2.07, 95%CI 1.21–3.54) showing a higher risk compared to group III (OR = 1.33, 95%CI 0.17–1.65). This underscores the importance of monitoring HPV infections to mitigate the risk of growth restrictions in fetuses^25^. Furthermore, neonates born to mothers with HPV co-infections have a significantly higher risk of requiring intensive care unit (ICU) admission (OR = 1.58, 95%CI 1.01–2.49). This finding emphasizes the need for vigilant prenatal care and potential early interventions to improve neonatal outcomes^26^.

Reflecting on the limitations of this study, several aspects warrant consideration. Firstly, despite the relatively large sample size of 7110 pregnant women, the study’s findings may still be limited by the cohort’s specific demographic and geographic characteristics, potentially affecting the generalizability of the results. Secondly, the study did not collect participants’ HPV vaccination information and the duration of infection with the HPV virus, which may have some influence on the interpretation of the findings. Future studies should systematically collect HPV vaccination data to further elucidate the effect of the vaccine on the study results. Continuous HPV testing is also needed to clarify the role of the time of infection. Thirtly, because the study was based on retrospective data, there was a lack of information on potentially confounding variables such as pre-pregnancy BMI, education, and economic status, which may have some impact on the comprehensiveness of the research results. Future studies need to collect relevant variables through an anticipatory design system to more rigorously validate the conclusions. Lastly, statistical capacity limitations due to the low incidence of some maternal and infant adverse outcomes. Future studies should further address the limitations of this study by expanding sample size, using large-scale multicenter prospective studies or mechanistic studies, and controlling for potential confounders to provide more reliable evidence of the relationship between HPV infection and maternal and infant health.

In conclusion, this study provides a comprehensive analysis of HPV infection distribution among pregnant women and its potential impact on pregnancy outcomes. These findings underscore the importance of HPV vaccination, thorough screening, and targeted management strategies to mitigate the risks associated with HPV infections during pregnancy. Further research should focus on elucidating the mechanisms underlying these associations and developing effective interventions to improve maternal and neonatal health outcomes.

## Supplementary Information

Supplementary Information.

## Data Availability

The datasets used and/or analysed during the current study available from the corresponding author on reasonable request.
