# Supplementary material for: The distribution and the relationship of HPV subtypes infection with pregnancy outcomes
Source: Sci Rep. 2025 Mar 24;15:10138. doi: 10.1038/s41598-025-94563-4 (PMC11933330; doi:10.1038/s41598-025-94563-4)
Supplement: Supplementary file 1 — Supplementary Information. [file 41598_2025_94563_MOESM1_ESM.docx]

**Supplementary Table 1 Effect of infection with HPV subtypes on maternal and neonatal outcomes**

| **Variable** | **group** | **n.total** | **n.event_%** | **Crude** | | **Adjusted** | | |  |
| --- | --- | --- | --- | --- | --- | --- | --- | --- | --- |
|  |  |  |  | **OR (95%CI)** | ***P*** value | **OR (95%CI)** | | ***P*** value |  |
| **GDM** | HPV group1 |  |  |  |  |  | |  |  |
|  | Control | 5690 | 1294 (22.7) | 1(Ref) |  | 1(Ref) | |  |  |
|  | I +II | 871 | 172 (19.7) | 0.84 (0.7~1) | 0.048 | 0.87 (0.72~1.04) | | 0.130 |  |
|  | III | 414 | 87 (21) | 0.9 (0.71~1.15) | 0.418 | 0.89 (0.7~1.15) | | 0.384 |  |
|  | IV | 135 | 27 (20) | 0.85 (0.55~1.3) | 0.453 | 0.92 (0.59~1.42) | | 0.704 |  |
|  | HPV group2 |  |  |  |  |  | |  |  |
|  | Control | 5690 | 1294 (22.7) | 1(Ref) |  | 1(Ref) | |  |  |
|  | I +III | 630 | 136 (21.6) | 0.94 (0.77~1.14) | 0.511 | 0.94 (0.77~1.15) | | 0.556 |  |
|  | II | 655 | 123 (18.8) | 0.79 (0.64~0.96) | 0.021 | 0.82 (0.66~1.01) | | 0.06 |  |
|  | IV | 135 | 27 (20) | 0.85 (0.55~1.3) | 0.453 | 0.92 (0.59~1.42) | | 0.703 |  |
|  | HPV group3 |  |  |  |  |  | |  |  |
|  | Control | 5690 | 1294 (22.7) | 1(Ref) |  | 1(Ref) | |  |  |
|  | I +II +III | 1285 | 259 (20.2) | 0.86 (0.74~1) | 0.044 | 0.88 (0.75~1.02) | | 0.094 |  |
|  | IV | 135 | 27 (20) | 0.85 (0.55~1.3) | 0.453 | 0.92 (0.59~1.42) | | 0.704 |  |
| **HDCP** | HPV group1 |  |  |  |  |  | |  |  |
|  | Control | 5690 | 187 (3.3) | 1(Ref) |  | 1(Ref) | |  |  |
|  | I +II | 871 | 39 (4.5) | 1.38 (0.97~1.96) | 0.074 | 1.42 (1~2.03) | | 0.051 |  |
|  | III | 414 | 13 (3.1) | 0.95 (0.54~1.69) | 0.872 | 0.95 (0.54~1.68) | | 0.860 |  |
|  | IV | 135 | 11 (8.1) | 2.61 (1.39~4.92) | 0.003 | 2.73 (1.44~5.15) | | 0.002 |  |
|  | HPV group2 |  |  |  |  |  | |  |  |
|  | Control | 5690 | 187 (3.3) | 1(Ref) |  | 1(Ref) | |  |  |
|  | I +III | 630 | 24 (3.8) | 1.17 (0.76~1.8) | 0.488 | 1.17 (0.76~1.81) | | 0.479 |  |
|  | II | 655 | 28 (4.3) | 1.31 (0.88~1.97) | 0.187 | 1.36 (0.91~2.05) | | 0.138 |  |
|  | IV | 135 | 11 (8.1) | 2.61 (1.39~4.92) | 0.003 | 2.73 (1.44~5.15) | | 0.002 |  |
|  | HPV group3 |  |  |  |  |  | |  |  |
|  | Control | 5690 | 187 (3.3) | 1(Ref) |  | 1(Ref) | |  |  |
|  | I +II +III | 1285 | 52 (4) | 1.24 (0.91~1.7) | 0.177 | 1.27 (0.92~1.73) | | 0.142 |  |
|  | IV | 135 | 11 (8.1) | 2.61 (1.39~4.92) | 0.003 | 2.72 (1.44~5.15) | | 0.002 |  |
| **PPROM** | HPV group1 |  |  |  |  |  | |  |  |
|  | Control | 5690 | 1448 (25.4) | 1(Ref) |  | 1(Ref) | |  |  |
|  | I +II | 871 | 258 (29.6) | 1.23 (1.05~1.44) | 0.009 | 1.23 (1.05~1.45) | | 0.010 |  |
|  | III | 414 | 94 (22.7) | 0.86 (0.68~1.09) | 0.215 | 0.86 (0.67~1.09) | | 0.203 |  |
|  | IV | 135 | 35 (25.9) | 1.03 (0.69~1.51) | 0.900 | 1.01 (0.68~1.49) | | 0.977 |  |
|  | HPV group2 |  |  |  |  |  | |  |  |
|  | Control | 5690 | 1448 (25.4) | 1(Ref) |  | 1(Ref) | |  |  |
|  | I +III | 630 | 152 (24.1) | 0.93 (0.77~1.13) | 0.469 | 0.92 (0.76~1.12) | | 0.424 |  |
|  | II | 655 | 200 (30.5) | 1.29 (1.08~1.54) | 0.005 | 1.29 (1.08~1.55) | | 0.005 |  |
|  | IV | 135 | 35 (25.9) | 1.03 (0.69~1.51) | 0.900 | 1.01 (0.68~1.49) | | 0.977 |  |
|  | HPV group3 |  |  |  |  |  | |  |  |
|  | Control | 5690 | 1448 (25.4) | 1(Ref) |  | 1(Ref) | |  |  |
|  | I +II +III | 1285 | 352 (27.4) | 1.11 (0.96~1.27) | 0.150 | 1.1 (0.96~1.27) | | 0.163 |  |
|  | IV | 135 | 35 (25.9) | 1.03 (0.69~1.51) | 0.900 | 1.01 (0.68~1.49) | | 0.977 |  |
| **Placental abruption** | HPV group1 |  |  |  |  |  | |  |  |
|  | Control | 5690 | 145 (2.5) | 1(Ref) |  | 1(Ref) | |  |  |
|  | I +II | 871 | 14 (1.6) | 0.62 (0.36~1.09) | 0.095 | 0.61 (0.35~1.07) | | 0.087 |  |
|  | III | 414 | 12 (2.9) | 1.14 (0.63~2.07) | 0.664 | 1.22 (0.67~2.22) | | 0.521 |  |
|  | IV | 135 | 3 (2.2) | 0.87 (0.27~2.76) | 0.812 | 0.96 (0.3~3.07) | | 0.951 |  |
|  | HPV group2 |  |  |  |  |  | |  |  |
|  | Control | 5690 | 145 (2.5) | 1(Ref) |  | 1(Ref) | |  |  |
|  | I +III | 630 | 15 (2.4) | 0.93 (0.54~1.6) | 0.800 | 0.97 (0.57~1.67) | | 0.918 |  |
|  | II | 655 | 11 (1.7) | 0.65 (0.35~1.21) | 0.177 | 0.64 (0.34~1.2) | | 0.164 |  |
|  | IV | 135 | 3 (2.2) | 0.87 (0.27~2.76) | 0.812 | 0.96 (0.3~3.07) | | 0.950 |  |
|  | HPV group3 |  |  |  |  |  | |  |  |
|  | Control | 5690 | 145 (2.5) | 1(Ref) |  | 1(Ref) | |  |  |
|  | I +II +III | 1285 | 26 (2) | 0.79 (0.52~1.2) | 0.273 | 0.8 (0.52~1.22) | | 0.303 |  |
|  | IV | 135 | 3 (2.2) | 0.87 (0.27~2.76) | 0.812 | 0.96 (0.3~3.06) | | 0.949 |  |
| **Postpartum** hemorrhage | HPV group1 |  |  |  |  |  | |  |  |
|  | Control | 5690 | 471 (8.3) | 1(Ref) |  | 1(Ref) | |  |  |
|  | I +II | 871 | 68 (7.8) | 0.94 (0.72~1.22) | 0.638 | 0.97 (0.75~1.27) | | 0.847 |  |
|  | III | 414 | 38 (9.2) | 1.12 (0.79~1.58) | 0.522 | 1.11 (0.78~1.57) | | 0.567 |  |
|  | IV | 135 | 9 (6.7) | 0.79 (0.4~1.57) | 0.502 | 0.84 (0.42~1.67) | | 0.618 |  |
|  | HPV group2 |  |  |  |  |  | |  |  |
|  | Control | 5690 | 471 (8.3) | 1(Ref) |  | 1(Ref) | |  |  |
|  | I +III | 630 | 56 (8.9) | 1.08 (0.81~1.44) | 0.599 | 1.08 (0.81~1.45) | | 0.591 |  |
|  | II | 655 | 50 (7.6) | 0.92 (0.68~1.24) | 0.570 | 0.95 (0.7~1.29) | | 0.760 |  |
|  | IV | 135 | 9 (6.7) | 0.79 (0.4~1.57) | 0.502 | 0.84 (0.42~1.67) | | 0.618 |  |
|  | HPV group3 |  |  |  |  |  | |  |  |
|  | Control | 5690 | 471 (8.3) | 1(Ref) |  | 1(Ref) | |  |  |
|  | I +II +III | 1285 | 106 (8.2) | 1 (0.8~1.24) | 0.973 | 1.02 (0.82~1.27) | | 0.875 |  |
|  | IV | 135 | 9 (6.7) | 0.79 (0.4~1.57) | 0.502 | 0.84 (0.42~1.67) | | 0.618 |  |
| **Cesarean delivery** | HPV group1 |  |  |  |  |  | |  |  |
|  | Control | 5690 | 2029 (35.7) | 1(Ref) |  | 1(Ref) | |  |  |
|  | I +II | 871 | 334 (38.3) | 1.12 (0.97~1.3) | 0.124 | 1.19 (1.03~1.39) | | 0.023 |  |
|  | III | 414 | 154 (37.2) | 1.07 (0.87~1.31) | 0.528 | 1.07 (0.86~1.33) | | 0.526 |  |
|  | IV | 135 | 59 (43.7) | 1.4 (0.99~1.98) | 0.055 | 1.59 (1.11~2.27) | | 0.011 |  |
|  | HPV group2 |  |  |  |  |  | |  |  |
|  | Control | 5690 | 2029 (35.7) | 1(Ref) |  | 1(Ref) | |  |  |
|  | I +III | 630 | 231 (36.7) | 1.04 (0.88~1.24) | 0.617 | 1.06 (0.88~1.26) | | 0.551 |  |
|  | II | 655 | 257 (39.2) | 1.17 (0.99~1.38) | 0.071 | 1.25 (1.06~1.49) | | 0.010 |  |
|  | IV | 135 | 59 (43.7) | 1.4 (0.99~1.98) | 0.055 | 1.59 (1.11~2.27) | | 0.011 |  |
|  | HPV group3 |  |  |  |  |  | |  |  |
|  | Control | 5690 | 2029 (35.7) | 1(Ref) |  | 1(Ref) | |  |  |
|  | I +II +III | 1285 | 488 (38) | 1.1 (0.97~1.25) | 0.118 | 1.15 (1.01~1.31) | | 0.031 |  |
|  | IV | 135 | 59 (43.7) | 1.4 (0.99~1.98) | 0.055 | 1.59 (1.11~2.27) | | 0.011 |  |
| **Premature birth** | HPV group1 |  |  |  |  |  | |  |  |
|  | Control | 5690 | 423 (7.4) | 1(Ref) |  | 1(Ref) | |  |  |
|  | I +II | 871 | 79 (9.1) | 1.24 (0.97~1.6) | 0.091 | 1.21 (0.93~1.58) | | 0.157 |  |
|  | III | 414 | 27 (6.5) | 0.87 (0.58~1.3) | 0.493 | 0.92 (0.62~1.38) | | 0.696 |  |
|  | IV | 135 | 12 (8.9) | 1.21 (0.67~2.22) | 0.526 | 1.35 (0.74~2.47) | | 0.328 |  |
|  | HPV group2 |  |  |  |  |  | |  |  |
|  | Control | 5690 | 423 (7.4) | 1(Ref) |  | 1(Ref) | |  |  |
|  | I +III | 630 | 42 (6.7) | 0.89 (0.64~1.24) | 0.484 | 0.91 (0.65~1.27) | | 0.572 |  |
|  | II | 655 | 64 (9.8) | 1.35 (1.02~1.78) | 0.034 | 1.32 (0.99~1.77) | | 0.058 |  |
|  | IV | 135 | 12 (8.9) | 1.21 (0.67~2.22) | 0.526 | 1.35 (0.74~2.47) | | 0.328 |  |
|  | HPV group3 |  |  |  |  |  | |  |  |
|  | Control | 5690 | 423 (7.4) | 1(Ref) |  | 1(Ref) | |  |  |
|  | I +II +III | 1285 | 106 (8.2) | 1.12 (0.9~1.4) | 0.319 | 1.11 (0.88~1.4) | | 0.357 |  |
|  | IV | 135 | 12 (8.9) | 1.21 (0.67~2.22) | 0.526 | 1.35 (0.74~2.47) | | 0.328 |  |
| **SGA** | HPV group1 |  |  |  |  |  | |  |  |
|  | Control | 5690 | 348 (6.1) | 1(Ref) |  | 1(Ref) | |  |  |
|  | I +II | 871 | 64 (7.3) | 1.21 (0.92~1.6) | 0.168 | 1.15 (0.87~1.52) | | 0.327 |  |
|  | III | 414 | 9 (2.2) | 0.34 (0.18~0.67) | 0.002 | 0.33 (0.17~0.65) | | 0.001 |  |
|  | IV | 135 | 16 (11.9) | 2.06 (1.21~3.51) | 0.008 | 2.07 (1.21~3.54) | | 0.008 |  |
|  | HPV group2 |  |  |  |  |  | |  |  |
|  | Control | 5690 | 348 (6.1) | 1(Ref) |  | 1(Ref) | |  |  |
|  | I +III | 630 | 29 (4.6) | 0.74 (0.5~1.09) | 0.131 | 0.71 (0.48~1.05) | | 0.088 |  |
|  | II | 655 | 44 (6.7) | 1.1 (0.8~1.53) | 0.554 | 1.05 (0.75~1.45) | | 0.789 |  |
|  | IV | 135 | 16 (11.9) | 2.06 (1.21~3.51) | 0.008 | 2.07 (1.21~3.54) | | 0.008 |  |
|  | HPV group3 |  |  |  |  |  | |  |  |
|  | Control | 5690 | 348 (6.1) | 1(Ref) |  | 1(Ref) | |  |  |
|  | I +II +III | 1285 | 73 (5.7) | 0.92 (0.71~1.2) | 0.551 | 0.88 (0.68~1.14) | | 0.344 |  |
|  | IV | 135 | 16 (11.9) | 2.06 (1.21~3.51) | 0.008 | 2.07 (1.21~3.54) | | 0.008 |  |
| **LGA** | HPV group1 |  |  |  |  |  | |  |  |
|  | Control | 5690 | 377 (6.6) | 1(Ref) |  | 1(Ref) | |  |  |
|  | I +II | 871 | 57 (6.5) | 0.98 (0.74~1.31) | 0.916 | 1.04 (0.78~1.39) | | 0.796 |  |
|  | III | 414 | 26 (6.3) | 0.95 (0.63~1.43) | 0.795 | 0.97 (0.64~1.47) | | 0.893 |  |
|  | IV | 135 | 11 (8.1) | 1.25 (0.67~2.33) | 0.489 | 1.28 (0.68~2.4) | | 0.446 |  |
|  | HPV group2 |  |  |  |  |  | |  |  |
|  | Control | 5690 | 377 (6.6) | 1(Ref) |  | 1(Ref) | |  |  |
|  | I +III | 630 | 32 (5.1) | 0.75 (0.52~1.09) | 0.137 | 0.78 (0.54~1.14) | | 0.202 |  |
|  | II | 655 | 51 (7.8) | 1.19 (0.88~1.61) | 0.269 | 1.25 (0.92~1.7) | | 0.155 |  |
|  | IV | 135 | 11 (8.1) | 1.25 (0.67~2.33) | 0.489 | 1.28 (0.68~2.4) | | 0.446 |  |
|  | HPV group3 |  |  |  |  |  | |  |  |
|  | Control | 5690 | 377 (6.6) | 1(Ref) |  | 1(Ref) | |  |  |
|  | I +II +III | 1285 | 83 (6.5) | 0.97 (0.76~1.24) | 0.824 | 1.02 (0.79~1.3) | | 0.893 |  |
|  | IV | 135 | 11 (8.1) | 1.25 (0.67~2.33) | 0.489 | 1.28 (0.68~2.4) | | 0.446 |  |
| **LBW** | HPV group1 |  |  |  |  |  | |  |  |
|  | | Control | 5690 | 325 (5.7) | 1(Ref) |  | 1(Ref) | |  |
|  | | I +II | 871 | 64 (7.3) | 1.31 (0.99~1.73) | 0.058 | 1.23 (0.91~1.65) | | 0.176 |
|  | | III | 414 | 21 (5.1) | 0.88 (0.56~1.39) | 0.587 | 0.94 (0.6~1.49) | | 0.802 |
|  | | IV | 135 | 11 (8.1) | 1.46 (0.78~2.74) | 0.233 | 1.63 (0.87~3.05) | | 0.130 |
|  | | HPV group2 |  |  |  |  |  | |  |
|  | | Control | 5690 | 325 (5.7) | 1(Ref) |  | 1(Ref) | |  |
|  | | I +III | 630 | 33 (5.2) | 0.91 (0.63~1.32) | 0.626 | 0.92 (0.63~1.34) | | 0.651 |
|  | | II | 655 | 52 (7.9) | 1.42 (1.05~1.93) | 0.023 | 1.35 (0.98~1.86) | | 0.068 |
|  | | IV | 135 | 11 (8.1) | 1.46 (0.78~2.74) | 0.233 | 1.63 (0.87~3.05) | | 0.130 |
|  | | HPV group3 |  |  |  |  |  | |  |
|  | | Control | 5690 | 325 (5.7) | 1(Ref) |  | 1(Ref) | |  |
|  | | I +II +III | 1285 | 85 (6.6) | 1.17 (0.91~1.5) | 0.214 | 1.13 (0.88~1.47) | | 0.342 |
|  | | IV | 135 | 11 (8.1) | 1.46 (0.78~2.74) | 0.233 | 1.63 (0.87~3.05) | | 0.130 |
| **[Macrosomia](https://context.reverso.net/translation/english-chinese/macrosomia)** | | HPV group1 |  |  |  |  |  | |  |
|  | | Control | 5690 | 250 (4.4) | 1(Ref) |  | 1(Ref) | |  |
|  | | I +II | 871 | 26 (3) | 0.67 (0.44~1.01) | 0.055 | 0.69 (0.46~1.05) | | 0.081 |
|  | | III | 414 | 13 (3.1) | 0.71 (0.4~1.24) | 0.227 | 0.72 (0.41~1.26) | | 0.247 |
|  | | IV | 135 | 7 (5.2) | 1.19 (0.55~2.57) | 0.658 | 1.2 (0.56~2.61) | | 0.639 |
|  | | HPV group2 |  |  |  |  |  | |  |
|  | | Control | 5690 | 250 (4.4) | 1(Ref) |  | 1(Ref) |  | |
|  | | I +III | 630 | 18 (2.9) | 0.64 (0.39~1.04) | 0.072 | 0.66 (0.4~1.07) | 0.089 | |
|  | | II | 655 | 21 (3.2) | 0.72 (0.46~1.13) | 0.156 | 0.74 (0.47~1.17) | 0.202 | |
|  | | IV | 135 | 7 (5.2) | 1.19 (0.55~2.57) | 0.658 | 1.2 (0.56~2.61) | 0.639 | |
|  | | HPV group3 |  |  |  |  |  |  | |
|  | | Control | 5690 | 250 (4.4) | 1(Ref) |  | 1(Ref) |  | |
|  | | I +II +III | 1285 | 39 (3) | 0.68 (0.48~0.96) | 0.028 | 0.7 (0.5~0.99) | 0.043 | |
|  | | IV | 135 | 7 (5.2) | 1.19 (0.55~2.57) | 0.658 | 1.2 (0.56~2.61) | 0.639 | |
| **NICU admission** | | HPV group1 |  |  |  |  |  |  | |
|  | | Control | 5690 | 706 (12.4) | 1(Ref) |  | 1(Ref) |  | |
|  | | I +II | 871 | 99 (11.4) | 0.91 (0.72~1.13) | 0.383 | 0.91 (0.73~1.14) | 0.418 | |
|  | | III | 414 | 51 (12.3) | 0.99 (0.73~1.34) | 0.958 | 1 (0.74~1.36) | 0.991 | |
|  | | IV | 135 | 24 (17.8) | 1.53 (0.98~2.39) | 0.064 | 1.58 (1.01~2.49) | 0.046 | |
|  | | HPV group2 |  |  |  |  |  |  | |
|  | | Control | 5690 | 706 (12.4) | 1(Ref) |  | 1(Ref) |  | |
|  | | I +III | 630 | 74 (11.7) | 0.94 (0.73~1.21) | 0.632 | 0.94 (0.73~1.22) | 0.655 | |
|  | | II | 655 | 76 (11.6) | 0.93 (0.72~1.19) | 0.553 | 0.94 (0.73~1.21) | 0.615 | |
|  | | IV | 135 | 24 (17.8) | 1.53 (0.98~2.39) | 0.064 | 1.58 (1.01~2.49) | 0.046 | |
|  | | HPV group3 |  |  |  |  |  |  | |
|  | | Control | 5690 | 706 (12.4) | 1(Ref) |  | 1(Ref) |  | |
|  | | I +II +III | 1285 | 150 (11.7) | 0.93 (0.77~1.13) | 0.469 | 0.94 (0.78~1.14) | 0.522 | |
|  | | IV | 135 | 24 (17.8) | 1.53 (0.98~2.39) | 0.064 | 1.58 (1.01~2.49) | 0.046 | |

Adjusted OR means adjusted for maternal age, Gravidity, Parity and Baby sex. **P*<0.05

GDM, gestational diabetes; HDCP, hypertensive disorder complicating pregnancy; PPROM, preterm premature rupture of membranes; SGA,small for gestational age; LGA, large for gestational age; LBW, low birth weight infant; NICU, neonatal intensive care unit;Control group means without HPV infection; group I means low risk HPV infection; group II means infection with nine-valent HPV vaccine covered high-risk HPV subtypes; group III means infection with non-nine-valent HPV vaccine covered high-risk HPV subtypes; group IV means co-infection with nine-valent HPV vaccine covered high-risk HPV subtypes and non-nine-valent HPV vaccine covered high-risk HPV subtypes.
